# Doping of Hollow Urchin-like MnO_2_ Nanoparticles in Beta-Tricalcium Phosphate Scaffold Promotes Stem Cell Osteogenic Differentiation

**DOI:** 10.3390/ijms26115092

**Published:** 2025-05-26

**Authors:** Enze Qian, Ahmed Eltawila, Yunqing Kang

**Affiliations:** 1 Department of Ocean & Mechanical Engineering, Florida Atlantic University, Boca Raton, FL 33431, USA; 2 Department of Biomedical Engineering, Florida Atlantic University, Boca Raton, FL 33431, USA; 3 Department of Biomedical Science, Florida Atlantic University, Boca Raton, FL 33431, USA; 4 Faculty of Integrative Biology PhD Program, Department of Biological Science, Florida Atlantic University, Boca Raton, FL 33431, USA; 5 Department of Chemistry and Biochemistry, Florida Atlantic University, Boca Raton, FL 33431, USA

**Keywords:** β-TCP scaffold, hollow MnO_2_ nanoparticles, hBMSC, biocompatibility, osteogenesis

## Abstract

Effective osteogenesis for bone regeneration is still considerably challenging for a porous β-tricalcium phosphate (β-TCP) scaffold to achieve. To overcome this challenge, hollow manganese dioxide (H-MnO_2_) nanoparticles with an urchin-like shell structure were prepared and added in the porous β-TCP scaffold. A template-casting method was used to prepare the porous H-MnO_2_/β-TCP scaffolds. As a control, solid manganese dioxide (S-MnO_2_) nanoparticles were also added into β-TCP scaffolds. Human bone mesenchymal stem cells (hBMSC) were seeded in the porous scaffolds and characterized through cell viability assay and alkaline phosphatase (ALP) assay. Results from in vitro protein loading and releasing experiments showed that H-MnO_2_ can load significantly higher proteins and release more proteins compared to S-MnO_2_ nanoparticles. When they were doped into β-TCP, MnO_2_ nanoparticles did not significantly change the surface wettability and mechanical properties of porous β-TCP scaffolds. In vitro cell viability results showed that MnO_2_ nanoparticles promoted cell proliferation in a low dose, but inhibited cell growth when the added concentration went beyond 0.5%. At a range of lower than 0.5%, H-MnO_2_ doped β-TCP scaffolds promoted the early osteogenesis of hBMSCs. These results suggested that H-MnO_2_ in the porous β-TCP scaffold has promising potential to stimulate osteogenesis. More studies would be performed to demonstrate the other functions of urchin-like H-MnO_2_ nanoparticles in the porous β-TCP.

## 1. Introduction

Rapid and efficient bone formation is still a considerable challenge for a tissue engineered β-tricalcium phosphate (β-TCP) scaffold. Tissue engineering strategies using β-TCP biomaterial scaffolds, cells, and growth factors have been developed to overcome the issues associated with autografts and allografts for bone tissue repairs [1,2,3,4,5]. However, translating tissue-engineered products into clinical applications is facing enormous difficulties and challenges. The progress achieved in laboratory studies has not yielded true benefits to clinical patients [6]. The reason may come from the following issues: classic stem-cell- or growth-factor-centric tissue regenerative strategies require seeding reproducible stem cells [7,8,9,10], maintaining the homogeneity of cultured cells in vitro [11,12,13], effectively directing stem cell differentiation, and ensuring a high survival rate and effective engraftment of seeded cells in vivo [14,15]. Obviously, it is extremely challenging for current biomaterial scaffolds to achieve the above-stated requirements.

For stimulating stem cell osteogenesis, many strategies have been studied; for example, using osteoinductive growth factor bone morphogenesis proteins (BMPs). However, speculations as to the safety and efficacy concerning the clinical application of BMPs remain, as a satisfactory level of data has not been generated yet, although the concern is based on the limited number of reports linking osteosarcomas with BMP activity [16,17]. Additionally, as BMPs are rapidly cleared from circulation by the liver, a well-characterized BMPs delivery carrier system is required to deliver BMPs in the implanted sites. Even so, 25 to 90% of the implanted dose after 3 h of implantation is lost, depending on the carriers [18,19]. Therefore, many studies explored alternative ways to add osteogenesis-stimulating materials into the scaffold to develop a new-generation bone substitute with excellent osteoinductivity and without the administration of any growth factor; for example, adding the elements magnesium [20], silicon [21], or strontium [22]. However, the effect of manganese ions (Mn^2+^) on osteogenesis seems to be overlooked, and has not been studied yet. Manganese is an essential trace element that is naturally present in many foods and available as a dietary supplement, and the human body contains about 10 to 20 mg manganese, of which 25% to 40% is in bone [23,24]. Manganese is a cofactor for several enzymes involved in bone formation [25]. In animals, manganese deficiency can impair bone formation and reduce bone mineral density [26], and manganese supplementation can increase both bone mineral density and bone formation [27].

Manganese dioxide (MnO_2_), an important and well-studied class of materials in catalysts, ion-exchangers, and batteries [28], has been proven to be biodegradable [29,30]. It displays excellent drug delivery capabilities, as it can react with the intracellular redox reagent glutathione (GSH), and produces vast amounts of water soluble Mn^2+^ ions [31,32,33]. Studies showed that MnO_2_-containing scaffolds promoted high survival rates, controlled differentiation of stem cells [34], and functional nerve recovery in a spinal cord injury animal model [35,36,37].

In our recent study [38], we successfully prepared hollow urchin-like MnO_2_ (H-MnO_2_) nanoparticles. The study results showed that hollow urchin-like MnO_2_ nanoparticles have higher surface area for drug loading, faster degradation rates for drug release, and improved colloidal stability, as compared to other potential drug delivery candidates, and they can be coated, loaded with a drug, and can target cells. However, whether the addition of such hollow urchin-like MnO_2_ nanoparticles into a β-TCP scaffold can enhance osteogenesis remains to be investigated. In this study, we added MnO_2_ nanoparticles in the porous β-TCP scaffold. We hypothesized that the addition of hollow urchin-like MnO_2_ nanoparticles in the porous β-TCP scaffold would significantly promote osteogenesis. To this end, solid and hollow MnO_2_ nanoparticles were doped into the β-TCP scaffolds. The porous β-TCP scaffolds with manganese dioxide nanoparticles were fabricated by our published method [39,40,41]. Human bone mesenchymal stem cells (hBMSC) were seeded on the scaffolds to characterize their behaviors through alkaline phosphatase (ALP) assay. The results showed that in a safety concentration range the MnO_2_/β-TCP scaffolds proved to have no adverse effects on the viability of hBMSCs and promoted their osteogenic differentiation and proliferation. These results suggested that the doping of MnO_2_ nanoparticles in the porous β-TCP scaffold potentially stimulated bone tissue regeneration.

## 2. Results

### 2.1. Morphology of H-MnO_2_ Nanoparticles

**Figure 1:**
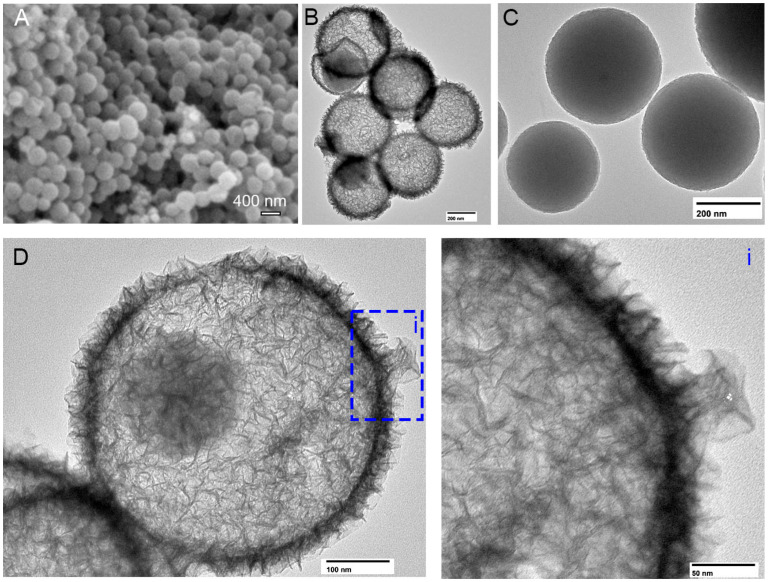
SEM photograph shows the morphologies of hollow MnO_2_ nanoparticles (**A**). TEM shows the hollow MnO_2_ nanoparticles (**B**), solid MnO_2_ nanoparticles (**C**), and the high magnified image of H-MnO_2_ nanoparticles (**D**) with a close view of the shell stings (**i**).

In this study, we prepared H-MnO_2_ nanoparticles first, and then added these nanoparticles into β-TCP for nanoparticle-doped scaffolds. The SEM image indicates that most of the nanoparticles have a similar size, which was determined by the diameter of the SiO_2_ template nanoparticles (Figure 1A). A TEM image clearly shows that the H-MnO_2_ nanoparticles have hollow structures (Figure 1B), but the S-MnO_2_ nanoparticles (US Research Nanomaterials, Inc., Houston, TX, USA) show a solid structure with a smooth surface (Figure 1C). Interestingly, the shell of H-MnO_2_ nanoparticles has many stings, which is mimetic to the shell of sea urchin (Figure 1D). The diameter of the nanoparticle is around 250 nm, with an around 25–40 nm shell (Figure 1D(i)).

### 2.2. Loading and Release Profile of the Proteins on H-MnO_2_ Nanoparticles

**Figure 2:**
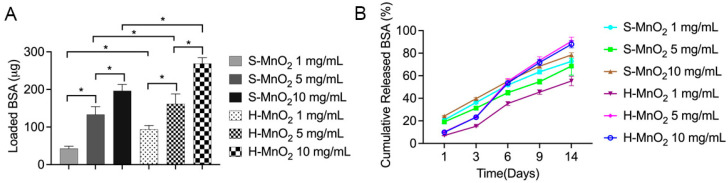
Loading amount of BSA on H-MnO_2_ nanoparticles and S-MnO_2_ nanoparticles (**A**), and their in vitro cumulative release (**B**) (The asterisk (*) represents a statistically significant difference) (*p* < 0.05).

To further confirm the function of such an urchin-like shell and hollow structure, protein loading and release experiments were performed. The results show that the hollow MnO_2_ nanoparticles can load significantly higher bovine serum albumin (BSA) proteins compared to S-MnO_2_ nanoparticles at the same addition of MnO_2_ nanoparticles mass (Figure 2A). Meanwhile, with the increase in the MnO_2_ nanoparticles (from 1 mg/mL to 10 mg/mL), regardless of hollow or solid nanoparticles, the BSA loading increased with the increased addition of MnO_2_ nanoparticles (Figure 2A). This result implies that the hollow structure can load more proteins than solid structure.

After we placed the BSA-loaded MnO_2_ nanoparticles into a phosphate-buffer saline (PBS) released medium, the released BSA was measured. The results show that both S-MnO_2_ and H-MnO_2_ nanoparticles can slowly release the adsorbed BSA with time (Figure 2B). Before 6 days, the H-MnO_2_ nanoparticles can slowly release less BSA from the nanoparticles compared to the S-MnO_2_ nanoparticles, and they can sustainably release BSA. This result implies that the hollow structure has better capacity that can not only load higher proteins, but also release more BSA continuously. This capacity may be related to the hollow urchin-like structure.

### 2.3. H-MnO_2_/β-TCP Scaffold Preparation

In this study, we added S-MnO_2_ and H-MnO_2_ nanoparticles into β-TCP to prepare MnO_2_-containing β-TCP scaffolds, and to evaluate their effect on the properties of the scaffolds. Various MnO_2_-doped β-TCP scaffold groups were created with MnO_2_ nanoparticles at weight concentrations of 0.25%, 0.5%, 1%, 3%, and 5% (*w*/*w*) for both H-MnO_2_ and S-MnO_2_.

**Figure 3:**
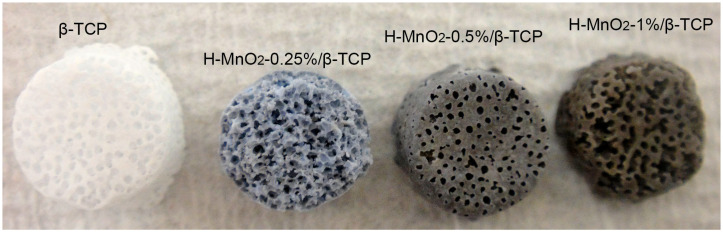
Digital images of porous β-TCP scaffolds: 0.25%, 0.5%, 1% H-MnO_2_ nanoparticle-doped β-TCP scaffolds.

We can see that the addition of MnO_2_ nanoparticles into β-TCP made the β-TCP scaffold change from white to black brown (Figure 3). With the increased addition of H-MnO_2_ nanoparticles, the black color of the H-MnO_2_/β-TCP becomes deeper. The diameter of the porous β-TCP scaffolds is around 7 mm, and the height is about 4 mm. This dimension was determined by the template. If the template was changed, the dimension of the resultant scaffold would be simultaneously changed.

**Figure 4:**
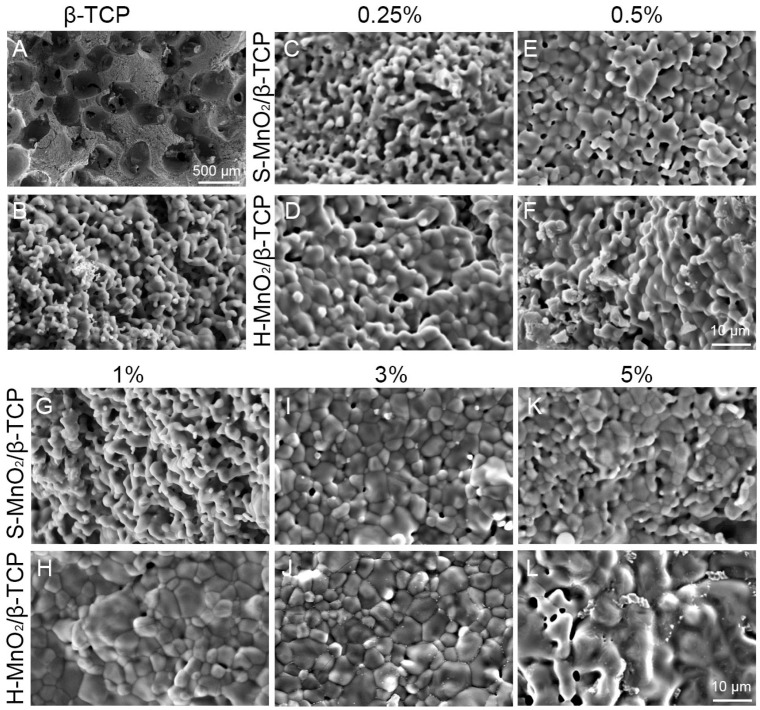
SEM images of porous β-TCP scaffolds (**A**,**B**), 0.25% S-MnO_2_ and H-MnO_2_ (**C**,**D**), 0.5% S-MnO_2_ and H-MnO_2_ (**E**,**F**), 1% S-MnO_2_ and H-MnO_2_ (**G**,**H**), 3% S-MnO_2_ and H-MnO_2_ (**I**,**J**), and 5% S-MnO_2_ and H-MnO_2_ doped β-TCP scaffolds (**K**,**L**), respectively. (Scale bar: **A**, 500 μm; **B**–**L**, 10 μm).

SEM images at a low magnification showed that the pores are interconnected, and the average pore size is about 400–450 μm (Figure 4A). The pore morphologies and porosity of these MnO_2_-containing β-TCP scaffolds are consistent with what we observed in the porous β-TCP scaffolds in the previous studies [39,40,41], as the preparation method for the scaffolds are the same as the ones used in our previous studies. From all cross-sections, the pores of the scaffolds are open to interconnect with each other, which could provide efficient paths for nutrient transportation and cell migrations. Further higher magnified SEM images showed that scaffolds without doped nanoparticles have a rough protruding structure (Figure 4B). However, as the percentage of MnO_2_ nanoparticles increases, the surface structure of inner struts became increasingly smooth, and the grain boundaries became blurry (Figure 4C–L). When the concentration of H-MnO_2_ nanoparticles increased to 5%, the grain boundary almost disappeared (Figure 4K,L).

### 2.4. Characterizations of the Scaffolds

**Figure 5:**
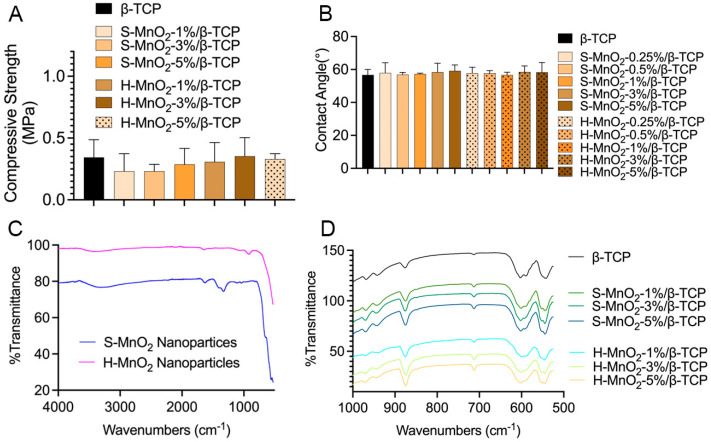
Compressive strength (**A**), contact angle (**B**), and FTIR (**C**,**D**) of porous β-TCP scaffolds, H-MnO_2_ nanoparticle-doped β-TCP scaffolds, and S-MnO_2_ nanoparticle-doped β-TCP scaffolds.

To investigate the effect of MnO_2_ nanoparticles on the mechanical properties of the porous scaffolds, both S-MnO_2_ and H-MnO_2_ nanoparticles with different doped concentrations were added into β-TCP scaffolds. The results show that the addition of S-MnO_2_ and H-MnO_2_ nanoparticles did not significantly influence the mechanical properties of the scaffolds (Figure 5A). There is no significant difference between those groups with different doped MnO_2_ nanoparticles.

Contact angle tests were performed to evaluate if the doping of H-MnO_2_ nanoparticles changes the hydrophilicity of the β-TCP scaffolds. The result shows there was no effect on the contact angle between the deionized water and the material. The contact angles of all types of scaffolds are about 57 degrees, which shows that the surface of all the scaffolds with the doped nanoparticles is hydrophilic, and no effect for the surface wettability was found (Figure 5B).

The FTIR analysis was performed to further characterize and confirm the possible effect of MnO_2_ on the chemical composition of the scaffolds (Figure 5C,D). The characteristic Mn-O vibration was expected at 550 cm^−1^ [42,43] (Figure 5C), which resulted in the appearance of a small duplet peak at 556 cm^−1^ in near position to the O-P-O bending peak at 550 cm^−1^ in the scaffold (Figure 5D). The absorption peak at around 1328.7 cm^−1^ is ascribed to O-Mn-O vibrations of MnO_2_ [44]. This peak in solid MnO_2_ nanoparticles is significant, but it disappeared in hollow MnO_2_ nanoparticles (Figure 5C). The disappearance could be derived of the hollow structure of H-MnO_2_ nanoparticles. The peaks at 550 and 605 cm^−1^ corresponds to the O-P-O bending vibration of β-TCP. The absorption band at around 720 cm^−1^ is due to the stretching vibrations of the oxide group of MnO_2_ in the form of Mn-O-Mn [45]. It was noted that, with the increase in MnO_2_ concentrations, the absorption band at around 720 cm^−1^ seems to became gradually more evident, and the hollow MnO_2_ groups have a more pronounced peak at 720 cm^−1^, compared to the solid MnO_2_ groups with the same concentration (Figure 5D).

### 2.5. Cell Viability and Osteogenesis

**Figure 6:**
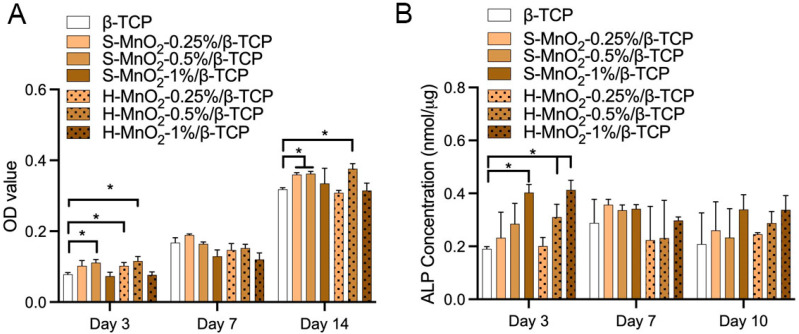
Cell viability (**A**) and ALP concentration (**B**) produced by hBMSCs on porous β-TCP scaffolds, H-MnO_2_ nanoparticle-doped β-TCP scaffolds and S-MnO_2_ nanoparticle-doped β-TCP scaffolds. (The asterisk (*) represents a statistically significant difference) (*p* < 0.05).

Figure 6A showed the result that the addition of MnO_2_ nanoparticles affected the cell growth. When β-TCP scaffolds contain 0.5% S-MnO_2_ and H-MnO_2_ nanoparticles, the doped nanoparticles promoted cell growth at day 3 and day 14. However, with the increased addition of S-MnO_2_ or H-MnO_2_ nanoparticles to 1%, the doped nanoparticles exhibited either no effect or inhibition to hBMSCs growth. It is worth noting that the hollow nanoparticles exhibit higher inhibition than the solid MnO_2_ nanoparticles when the mass ratio of MnO_2_ nanoparticles was higher than 1%, but it seems that 0.5% H-MnO_2_ nanoparticles promoted higher cells growth compared to 0.5% S-MnO_2_ group. Beyond 1%, including 3% and 5%, regardless of hollow or solid MnO_2_ nanoparticle-doped groups, there was a significant inhibition on cell growth (Supplementary Figure S1). These results seem that there is no consistent trend in the cell growth rate in the different MnO_2_ nanoparticle-doped scaffolds. However, we can still see that 0.5% doped concentration is a peak.

ALP activity measurement showed that at day 3, both 0.5% and 1% H-MnO_2_ nanoparticle-doped scaffolds had significantly higher ALP levels compared to β-TCP scaffolds, but only 1% S-MnO_2_ nanoparticle-doped β-TCP scaffolds show a stimulated effect (Figure 6B). This result implies that the H-MnO_2_ nanoparticles may be more effective to promote osteogeneses of hBMSCs. At day 7 or 14, the significant difference between all groups disappeared.

## 3. Discussion

Enormous studies have been using β-TCP scaffolds for bone tissue engineering applications. However, the ideal β-TCP scaffold has not been achieved yet. In this study, porous β-TCP scaffolds were doped with different concentrations of either hollow or solid MnO_2_ nanoparticles (0.25%, 0.5%, 1%, 3% or 5%) to achieve the osteogenesis-stimulating function. Studies showed that MnO_2_ have free radical scavenging effect [46], which makes MnO_2_ an attractive doping material for bone regeneration after infection or cancer therapy [47]. However, the in vitro interaction between hBMSCs and MnO_2_-doped β-TCP scaffolds had not been well investigated.

In this study, we used SiO_2_ as a template to prepare hollow MnO_2_ nanoparticles, and the H-MnO_2_ nanoparticles showed a hollow structure resembling a sea urchin, which increases the surface area and accelerates the degradation of manganese dioxide [38]. Regarding the effect of the addition of such hollow MnO_2_ nanoparticles into β-TCP scaffolds on the morphological properties of the scaffolds, the surface structure of the scaffolds became increasingly smooth as the percentage of MnO_2_ nanoparticles increases (Figure 4). It is likely that, during the sintering process, the MnO_2_ promoted the sintering of β-TCP granules to be densified. It is worth noting that, at low concentrations of MnO_2_ doping (0.25, 0.5, and 1%), hollow MnO_2_ groups also had a smoother surface than solid MnO_2_-doped scaffold groups. This phenomenon could be due to the larger surface area of the hollow nanoparticles, which can promote the migration and densification of the β-TCP particles during the sinter process. The real reasons remain unknown, as there was no intended experiment set in this study to investigate the sintering mechanisms. However, it is worth noting that the hollow structure of H-MnO_2_ nanoparticles may still be maintained in the β-TCP scaffold, although it looks like “melting”. This is because the early osteogenesis result (Figure 6B) showed that the H-MnO_2_-doped scaffolds promoted early osteogenesis compared to S-MnO_2_-doped scaffolds, which indirectly implied that the hollow structure may still be maintained in the porous β-TCP scaffolds during the sintering process to take effects on osteogenesis.

The contact angle was used to verify whether the change in microstructure of a hollow structure affected the surface free energy of the β-TCP scaffold. As samples with porous structures are challenging to test through the contact angle settings, the MnO_2_-doped β-TCP composite materials were prepared into a dense disk-like shape to represent the surface of a porous scaffold. We found that the doping of MnO_2_ did not change the surface hydrophilicity of all the groups of MnO_2_-doped β-TCP disks compared to β-TCP (Figure 5B). Based on this result, we theoretically assume that the addition of MnO_2_ nanoparticles did not change the surface hydrophilicity of the porous MnO_2_-doped β-TCP scaffolds compared to the porous β-TCP scaffold. However, the addition of MnO_2_ made the surface of the porous MnO_2_-doped β-TCP scaffolds rougher compared to the β-TCP scaffold (Figure 4) [48,49].

Regarding the biological properties of the scaffolds, the cell viability experiment showed that scaffolds with less than 1% MnO_2_ content are biocompatible, and groups that contain high hollow nanoparticles exhibited a greater inhibition on cell growth. This greater inhibition may be due to the faster degradation of hollow particles, which cause the faster release of the Mn ions. More Mn ions may induce toxicity. This assumed reason may need to be verified as we did not perform a degradation test on the scaffold in this study, but the degraded rate of H-MnO_2_ nanoparticles was faster than S-MnO_2_ nanoparticles in our previously published study [38].

For further investigating the effect of MnO_2_ nanoparticles on cell proliferation and differentiation of hBMSC, we analyzed the cell growth on those scaffolds. We found that the addition of MnO_2_ nanoparticles significantly affected cell proliferation, especially in a high-doped MnO_2_ scaffold group. More surprisingly, this effect is more significant in hollow MnO_2_-doped scaffolds when the doping increased to beyond 1%. This result may imply that there may be a threshold of doped concentration of MnO_2_ nanoparticles. These results seemed to be consistent with F. Qian’s study [42], where they found that with the increase of MnO_2_ to 0.5%, the cell proliferation was inhibited. On the other hand, ALP results demonstrated that 0.5% H-MnO_2_ nanoparticle-doped scaffold promoted early osteogenic differentiation of hBMSCs (at day 3), while S-MnO_2_ nanoparticles did not at this doped concentration until it increased 1%, although the difference in ALP level at both day 7 and 14 was statistically insignificant between all test groups. This result implied that hollow structure promoted both proliferation and differentiation when it was doped at a low dose (higher than 0.25% but less than 0.5%). At present, there are few studies on the effect of doped hollow MnO_2_ nanoparticles on early osteogenesis. Many studies are exploring the use of MnO_2_ nanoparticles for scavenging superoxide’s radicals in inflammatory conditions, such as arthrosclerosis [50], cancers, and cytoprotection of pancreatic islets of Langerhans in vitro [51]. For stimulating the osteogenesis of bone marrow stem cells for bone regeneration, to the best of our knowledge, very few studies show that solid MnO_2_ nanoparticles in a hydrogel or Mn-containing ceramic promoted the repair of osteoporotic bone defects [52,53]. However, little research has been performed on urchin-like hollow structure of MnO_2_ nanoparticles for bone regeneration. In this study, we doped hollow MnO_2_ nanoparticles into β-TCP scaffolds. This doping brings a unique function, which has not only the osteoconductivity of β-TCP and the porous structure of the scaffold, but also the osteogenesis-promoting function of hollow-structured MnO_2_ nanoparticles.

Although there are many unique advantages of this new structured MnO_2_ nanoparticle-doped β-TCP scaffold, some limitations in this study exist; for example, there is no test on the effect of doped MnO_2_ nanoparticles on the scavenging of radicals like H_2_O_2_ that was performed in [42]. It is also worth noting that the integrity of the hollow structure of H-MnO_2_ nanoparticles after doping in β-TCP scaffolds was not verified due to the technical challenges by SEM. As the doped concentration of nanoparticles in the scaffolds was less than 1–5%, it is challenging for SEM to identify them. Using protein loading and release profiles on the nanoparticles before doping, and on the scaffolds after doping, may be ways to indirectly prove the integrity of the hollow structure. Investigating the radical-scavenging function of the nanoparticles before doping and the scaffolds after doping could also be another indirect approach to verify the structural integrity and related functions. Another limitation in this study is that there was no further characterization on how hollow MnO_2_ nanoparticles affects cell behaviors including cell morphologies, osteogenic protein expression, and bone-related gene expressions. The related mechanisms that hollow MnO_2_ nanoparticles stimulate osteogenesis and potentially scavenge bone defect-related reactive oxygen species radicals [54] were not explored in this study. These functional and mechanistic studies will be performed in the future.

Even though these limitations, this study successfully provided a preliminary data on the preparation of urchin-like hollow structured nanoparticles in the β-TCP scaffold and the maximum doped concentration of H-MnO_2_ nanoparticles, which built the foundation for the future studies. Our current study brought a new potential of using the urchin-like hollow structure for bone tissue regeneration applications.

## 4. Materials and Methods

### 4.1. Materials

β-TCP nanopowders were purchased from Nanocerox (Ann Arbor, MI, USA). Magnesium acetate was purchased from Sigma-Aldrich (St. Louis, MO, USA). Carboxymethyl cellulose powder, dispersant (Darvan C), and surfactant antifoam solution (Surfonals) were obtained from Fisher Scientific (Waltham, MA, USA). Solid MnO_2_ (S-MnO_2_) nanoparticles were obtained from US Research Nanomaterials, Inc. (Houston, TX, USA). Mesenchymal Stem Cell Growth Kit (MSCGM) was purchased from ATCC (Manassas, VA, USA).

### 4.2. Preparation and Characterization of H-MnO_2_ Nanoparticles

Hollow MnO_2_ nanoparticles were fabricated using an SiO_2_ template [38,55]. Briefly, 100 mL of ethanol, 20 mL of deionized water, and 2 mL of 28% ammonia were mixed in a beaker with continuous stirring. A total of 8 mL of tetrapropyl orthosilicate (Fisher Scientific, Waltham, MA, USA) were then added to the mixture solution, keeping stirring for overnight. The milky solution was centrifuged at 6000 rpm for 20 min. The supernatant was discarded, and the precipitated pellets were washed three times with deionized water. The final precipitate pellets were then resuspended in 60 mL of DI water and mixed with 1.96 g of potassium permanganate (Sigma-Aldrich). The suspension was sonicated for 30 min, and then transferred into a Teflon-lined autoclave inside an oven. The suspension was incubated in the oven for 48 h at 150 °C. After cooling down, the mixture was centrifuged and washed with water until the purple color was clear. A solution of 300 mL of 2 M sodium carbonate (Fisher Scientific) was then added to etch the template SiO_2_ nanoparticles at 60 °C. To completely etch the template SiO_2_ nanoparticles, up to 2 days’ incubation in the sodium carbonate solution was carried out. The final hollow MnO_2_ nanoparticles were obtained after several times of washing, and then freeze-dried.

The dried H-MnO_2_ nanoparticles were observed by scanning electron microscope (SEM, JCM-6000Plus, Tokyo, Japan). To prepare the samples for the SEM observation, H-MnO_2_ nanoparticles were tapped on a SEM holder stage, and then coated with gold in a Pelco SC-7 sputter coater (Leica, Teaneck, NJ, USA).

To further measure the size and observe the detailed morphology, H-MnO_2_ nanoparticles were prepared for transmission electron microscopy (TEM, JEM-1400Flash, Tokyo, Japan). Briefly, a few milligrams of nanoparticles were dispersed in an ethanol solution, and the suspension was then dropped on a Formvar/Carbon 200 mesh. After drying, the holder was loaded to the TEM with 120 kV of acceleration voltage for imaging.

To show the function of hollow structure of the MnO_2_ nanoparticle in loading proteins, a model protein bovine serum albumin (BSA) (Fisher Scientific) was used. Different mass of hollow MnO_2_ and S-MnO_2_ nanoparticles were weighed and added into a BSA solution to load BSA. The concentration of the BSA solution is 100 μg/mL. The nanoparticles were added into the BSA solutions for 24 h at room temperature on a rotator with 30 rpm. At the end of the time, the BSA solution was collected and measured by Micro BCA™ Protein Assay Kit (Thermofisher Scientific, Waltham, MA, USA) and the optical density (OD) value was read by a SpectraMax 190 microplate reader (Molecular Devices LLC, San Jose, CA, USA) at the wavelength of 562 nm. The loading amount was obtained from the difference between masses before and after adsorption of BSA proteins. After 24 h, the nanoparticles were centrifuged and collected for in vitro release. The released BSA was calculated and the cumulative released mass of BSA was profiled against the time.

### 4.3. Preparation of MnO_2_/β-TCP Scaffolds

To prepare H-MnO_2_/β-TCP and S-MnO_2_/β-TCP scaffolds, different doped weight of MnO_2_ were set with MnO_2_ nanoparticles at weight concentrations of 0.25%, 0.5%, 1%, 3%, and 5% (*w*/*w*) for both H-MnO_2_ and S-MnO_2_. A template-casting method, as previously described, was used to prepare H-MnO_2_/β-TCP and S-MnO_2_/β-TCP scaffolds [41]. Briefly, a 24-well plate was coated with paraffin solution, then paraffin beads with 1 mm diameter were packed into the wells. β-TCP nanopowder from Nanocerox (Ann Arbor, MI, USA), dispersant (Darvan C), antifoam solution, magnesium acetate and MnO_2_ nanoparticles were mixed in distilled water to form a MnO_2_/β-TCP slurry. The mixed MnO_2_/β-TCP slurry was then cast into the molds under vacuum, solidified in 70% ethanol for two days, and then dehydrated by gradient ethanol from 70% to 95%. After complete dehydration, the green bodies were sintered at 1250 °C for 3 h. Porous MnO_2_/β-TCP scaffolds with different concentrations of S-MnO_2_ and H-MnO_2_ were prepared and stored for physicochemical and biological characterizations.

### 4.4. Characterization of the Porous Scaffolds

#### 4.4.1. SEM Observations

The surface and pore morphologies of porous scaffolds were observed by scanning electron microscope (SEM, JCM-6000Plus, Tokyo, Japan). Briefly, the scaffolds were washed by ethanol, and air-dried in a fume hood. The dried scaffolds were cut longitudinally or transversely and then taped on a SEM stage, and coated with gold in a Pelco SC-7 sputter coater. The scaffolds were observed under a voltage of 15 KV.

#### 4.4.2. Contact Angle Measurement

In order to reduce the influence of the porous structure on the contact angle measurement, the MnO_2_/β-TCP composites were specially prepared to a thin disk shape. An Ossila contact angle measurement system (South Yorkshire, UK) was used to test the contact angle between the material and the DI water. The raw images were analyzed by the software and output the angle data automatically.

#### 4.4.3. FTIR Measurement

The chemical structure of scaffolds was analyzed by Fourier transform infrared (FTIR). MnO_2_ nanoparticles, β-TCP, S-MnO_2_/β-TCP and H-MnO_2_/β-TCP scaffolds were tested through the Thermo Scientific Nicolet iS10 ATR-FTIR spectrometer (Waltham, MA, USA) to identify the chemical groups. The dried scaffolds were grounded to powders and made into a thin film. FTIR spectra was collected with 20 scans at 4.0 cm^−1^ resolution.

#### 4.4.4. Compressive Strength of Scaffolds

To measure the compressive strength of the scaffolds, a Zwick-Roell universal tension-compression machine Z50 (Ulm, Germany) was used following the American Society for Testing and Materials (ASTM) standards. To ensure the two ends of the scaffolds were parallel to the crosshead of the testers, the top and bottom sides of the scaffolds were polished using a LANHU 600 Grit electro coated abrasive sandpaper obtained from Amazon (Seattle, WA, USA). The diameter of each scaffold was individually measured. A crosshead speed of 0.5 mm/min was applied to each scaffold until fracture. Five samples per group were measured.

### 4.5. hBMSC Cell Behaviors

#### 4.5.1. hBMSC Culture

hBMSCs were purchased from ATCC (Manassas, VA, USA). The cells were cultured with Mesenchymal Stem Cell Growth Kit (MSCGM, ATCC, USA) under a standard condition (5% CO_2_, 95% humidity, and 37 °C). The cells at the passages 4–7 were used for all of the experiments.

#### 4.5.2. Cell Proliferation on the Scaffolds

A total of 5 × 10^4^ cells in 100 µL cell suspension were pre-seeded on the porous scaffold for 90 min, and MSCGM medium was gently added to incubate the cells in a 24-well plate at 37 °C. After 3, 7, and 14 days, the porous scaffolds were washed by PBS and transferred to a new well plate. A PBS with 10% MTT was added and incubated for 4 h. The medium was then carefully removed, and dimethyl sulfoxide (DMSO) was added to dissolve the formazan. Repeatedly the scaffolds were rinsed by DMSO during the process to ensure that all formazan crystals were dissolved. All of the solutions were collected and centrifuged, and the supernatant DMSO was then transferred to a 96-well plate. The OD value was read by plate reader at the wavelength of 490 nm.

#### 4.5.3. Alkaline Phosphatase (ALP)

ALP activity was evaluated to investigate the effect of MnO_2_ on potential osteogenic differentiation of hBMSCs. The cells were cultured with scaffolds in an osteogenic differentiation medium, which contained 10% FBS, 10 mM β-glycerophosphate, 10 nM dexamethasone, and 50 mg/mL ascorbic acid in the MCSBM. The hBMSCs were continuously cultured for 3, 7, and 14 days. After rinsing by PBS, the cells and scaffolds were collected and stored in −80 °C. To determine the ALP activity quantitatively, the total protein and total ALP were measured based on the published protocols [40]. Briefly, cell lysate of hBMSCs was prepared. The total protein in the hBMSCs cell lysate was measured by Micro BCA™ Protein Assay Kit (Thermofisher Scientific), and the OD value was read by a SpectraMax 190 microplate reader at the wavelength of 562 nm. The ALP was determined by a p-nitrophenyl phosphate (pNPP) method following our published protocol [56]. SIGMAFAST™ p-Nitrophenyl phosphate Tablets (Millipore Sigma, St. Louis, MO, USA) were used to prepare the working reagent and incubated at 37 °C for 3 h. The OD value was read by a plate reader at the wavelength of 405 nm. The final total ALP activity was normalized by the total protein contents of each sample.

### 4.6. Statistical Analysis

All of the collected data were analyzed using unpaired *t*-tests or one-way analyses of variance (ANOVA) with GraphPad Prism 7 (GraphPad, Boston, MA, USA), and *p* < 0.05 was considered statistically significant.

## 5. Conclusions

In this study, we successfully prepared urchin-like hollow MnO_2_ nanoparticles and then doped them into β-TCP scaffolds to make hollow MnO_2_ nanoparticle-doped β-TCP scaffolds. The scaffold shows interconnected pores, and H-MnO_2_ nanoparticles enhanced the sintering of β-TCP scaffolds. The addition of hollow MnO_2_ nanoparticles did not significantly change the mechanical properties and surface hydrophilicity of the scaffolds. The low dose H-MnO_2_-doped β-TCP scaffolds promoted the cell proliferation and early osteogenesis of human mesenchymal stem cells. In the whole, H-MnO_2_ nanoparticles demonstrated more advantages in promoting cell growth and osteogenesis compared to solid MnO_2_ nanoparticles when they were doped into the β-TCP scaffolds at a low concentration. More studies would be needed to further investigate the function and mechanisms of H-MnO_2_ in bone regeneration.

## Data Availability

Data will be made available on request.

## Supplementary Materials

The following supporting information can be downloaded at: https://www.mdpi.com/article/10.3390/ijms26115092/s1.
