# Supplementary material for: Doping of Hollow Urchin-like MnO2 Nanoparticles in Beta-Tricalcium Phosphate Scaffold Promotes Stem Cell Osteogenic Differentiation
Source: Int J Mol Sci. 2025 May 26;26(11):5092. doi: 10.3390/ijms26115092 (PMC12154069; doi:10.3390/ijms26115092)
Supplement: Supplementary file 1 [file ijms-26-05092-s001.zip › ijms-3580391-supplementary.pdf]

**Supplementary Materials**

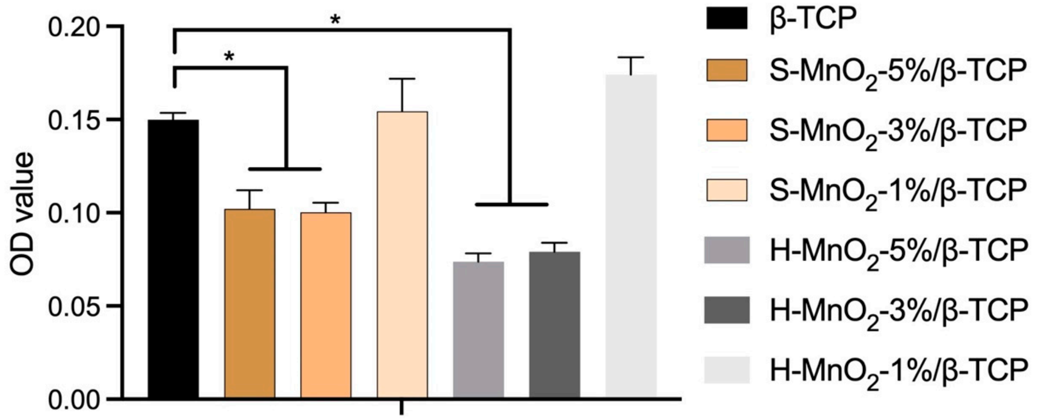


**Figure S1.** Cell viability on S-$MnO_2$ and H-$MnO_2$ doped β-TCP scaffolds. (The asterisk (*) represents a statistically significant difference) ($p < 0.05$).
